# Safflower (*Carthamus tinctorius* L.) crop adaptation to residual moisture stress: conserved water use and canopy temperature modulation are better adaptive mechanisms

**DOI:** 10.7717/peerj.15928

**Published:** 2023-09-11

**Authors:** Chennamsetti Manikanta, Ratnakumar Pasala, Sivasakthi Kaliamoorthy, P. S. Basavaraj, Brij Bihari Pandey, Dinesh Rahul Vadlamudi, Mukta Nidamarty, Arti Guhey, Palchamy Kadirvel

**Affiliations:** 1 ICAR-Indian Institute of Oilseeds Research, Rajendranagar, Hyderabad, India; 2 Indira Gandhi Agricultural University, Raipur, Chhattisgarh, India; 3 ICRISAT-International Crops Research Institute for the Semi-Arid Tropics Patancheru, Greater Hyderabad, Telangana, India; 4 ICAR-National Institute of Abiotic Stress Management, Baramati, Maharashtra, India

**Keywords:** Safflower, Soil moisture depletion, Transpiration efficiency, Canopy temperature depression

## Abstract

Oilseeds with high productivity and tolerance to various environmental stresses are in high demand in the food and industrial sectors. Safflower, grown under residual moisture in the semi-arid tropics, is adapted to moisture stress at certain levels. However, a substantial reduction in soil moisture has a significant impact on its productivity. Therefore, assessing genetic variation for water use efficiency traits like transpiration efficiency (TE), water uptake, and canopy temperature depression (CTD) is essential for enhancing crop adaptation to drought. The response of safflower genotypes (*n* = 12) to progressive soil moisture depletion was studied in terms of water uptake, TE, and CTD under a series of pot and field experiments. The normalised transpiration rate (NTR) in relation to the fraction of transpirable soil water (FTSW) varied significantly among genotypes. The genotypes A-1, Bhima, GMU-2347, and CO-1 had higher NTR-FTSW threshold values of 0.79 (R^2^ = 0.92), 0.74 (R^2^ = 0.96), 0.71 (R^2^ = 0.96), and 0.71 (R^2^ = 0.91), respectively, whereas GMU-2644 had the lowest 0.38 (R^2^ = 0.93). TE was high in genotype GMU-2347, indicating that it could produce maximum biomass per unit of water transpired. At both the vegetative and reproductive stages, significant positive relationships between TE, SPAD chlorophyll metre reading (SCMR) (*p* < 0.01) and CTD (*p* < 0.01) were observed under field conditions by linear regression. The genotypes with high FTSW-NTR thresholds, high SCMR, and low CTD may be useful clues in identifying a genotype’s ability to adapt to moisture stress. The findings showed that the safflower genotypes A-1, Bhima, GMU-2347, and CO-1 exhibited an early decline and regulated water uptake by conserving it for later growth stages under progressive soil water depletion.

## Introduction

Safflower is an important oilseed crop grown for its edible oil, which is composed of 90% unsaturated fatty acids, *i.e*., oleic and linoleic acids (Johnson, Bergman & Flynn, 1999). Though safflower is a crop with xeric adaptations to water stress, a delay in sowing or a decline in soil available water often leads to drought stress (Yau, 2007). The severity of water stress is expected to increase in the near future given the conditions of climate change, mainly characterised by long periods of null precipitation rate or shorter windows of intense precipitation (Minhas, Rane & Pasala, 2017). “Drought” is defined as a scenario where the available water is insufficient to meet the transpiration demand of the plant. An increase in evapotranspiration rate further driven by global warming aggravates the frequency of drought incidence (Rahmani et al., 2019). Despite the ability of the safflower crop to withstand water stress, there still exists a significant difference among the safflower genotypes in response to terminal water stress. Drought curtails a genotype’s ability to achieve reliable yields. Genotypes that are able to “produce more crop per drop” are expected to have high transpiration efficiency (TE), *i.e*., the ratio of plant dry matter produced for every unit amount of water transpired (Ratnakumar et al., 2009; Geetika et al., 2019). High transpiration efficiency in crop plants is an adaptive mechanism to increase yields under rainfed conditions where reduced water supply limits biomass production (Katerji, Mastrorilli & Rana, 2008; Ratnakumar & Vadez, 2011; Christy et al., 2018; Ratnakumar et al., 2021).

Water relations are under the influence of certain factors, which includes leaf water status, canopy temperature, stomatal conductance, and transpiration rate (Fahad et al., 2017). Of all the physiological processes, water stress impairs the gaseous exchange rate by stomatal closure which ultimately results in reduced carbon dioxide assimilation. Genotypes differ among themselves in responding to prevailing soil moisture conditions. Certain genotypes tend to regulate transpiration rate at whole plant level in the early stages of growth even the soil is relatively moist anticipating severe stress during later crucial stages, *i.e*., the reproductive period, whereas a few may fail to monitor and may experience the loss of water within a short window of stress incidence (Edwards, Ewers & Weinig, 2016). During the soil drying process, there exists a threshold or break point (bp) (identified using the fraction of transpirable soil water, *i.e*., FTSW) prior to which soil moisture, the plant transpiration is maximum and thereafter a decline in soil moisture starts influencing plant transpiration. Hence, the FTSW-NTR threshold point is employed as an indicator in explaining the genotypic differences for TE (Devi et al., 2009). Though in recent past, studies aimed in identifying morpho-physiological, yield related (Khalili et al., 2014) and biochemical traits (Javed et al., 2013) in conferring tolerance to water stress, identifying threshold point at which a genotype starts experiencing stress will be a more precise way in categorizing genotypic response to stress. This kind of information is lacking in safflower. Similar experiments were conducted in various crops, *i.e*., soybean (Liu et al., 2005), groundnut (Devi et al., 2009; Vadez & Ratnakumar, 2016), wheat (Faralli et al., 2019), maize (Ray et al., 2002), cowpea (Belko et al., 2012), chickpea (Sivasakthi et al., 2019) and rice (Heinemann, Stone & Fageria, 2011).

Another aspect of water relations under stress scenario is the genotypic ability to regulate water loss at the leaf level. A conservative pattern in water utilisation always helps the crop to tide over terminal drought by maintaining moisture levels in the soil profile for later stages, as reported in pearl millet (Kholová et al., 2010), chickpea (Zaman-Allah, Jenkinson & Vadez, 2011b) soybean (Bagherzadi et al., 2017) and sorghum (Devi & Reddy, 2020).

Though genetic variation for TE among the genotypes can be well elucidated with pot experiments, extrapolating it to regular breeding programmes under field conditions is a bottleneck, which emphasises the role of surrogate traits. TE under field conditions is often related to easy and non-destructive traits such as SPAD chlorophyll metre reading (SCMR), specific leaf area (SLA) (Nigam & Aruna, 2008; Devi et al., 2011; Pandey et al., 2021) and canopy temperature depression (CTD) (Royo et al., 2002). Evaluating these traits under field conditions could serve as a reliable index for TE. It can be hypothesized that the identification of soil threshold at which normal transpiration gets affected, TE and associated surrogate traits could be an effective screening tools in combating water stress. In this context, the objectives of the present study were (i) to estimate and categorize the threshold FTSW-NTR values at which the safflower genotype starts regulating transpiration and (ii) understand the importance of surrogate traits viz., SPAD chlorophyll meter reading (SCMR) and canopy temperature depression as a selection criteria for TE under field conditions.

## Material and Methods

Pot and field experiments were conducted during the post-rainy season for two consecutive years, 2020–21 and 2021–22. Pot studies were carried out in a completely randomised design (CRD) with six replications of plants for control and progressive water-stressed, whereas the field experiments were conducted under randomised complete block (RBD) design with three replications. Based on previous studies, where genotypes were evaluated under water deficit conditions at different locations, 12 genotypes of safflower comprising germplasm and released varieties were selected (Table S1).

### Experiment-1: evaluation of safflower genotypes for transpiration under progressive soil moisture stress

For measuring transpiration using gravimetric method among the genotypes, pot experiments were conducted at ICAR-Indian Institute of Oilseeds and Research, Rajendranagar, Hyderabad (17.32012°N, 78.40931°E, and 487 m above MSL). Pots were filled with 12 kg of black soil (Order: Vertisol) and five seeds were sown in each pot during the last week of October. After the successful establishment of the crop, the plants were thinned and only two healthy plants were retained. At 45 days after sowing (DAS), when the plants were at the vegetative stage, pots were irrigated until saturation and left drained for overnight to remove the excess moisture. At this point, the soil in the pots was at 100 per cent field capacity. Before the onset of different water regimes, a replication set from the control as well as water-stressed pots were harvested and the initial biomass was recorded. The soil in pots was covered with a polypropylene cover to avoid evaporation. An approximate 400 g of filler, plastic beads were spread over to reduce soil temperature, leaving transpiration as the only way to lose water from the soil. The pots were weighed and recorded the initial weights. Later on, daily wise, the pots were weighed at 8:00 AM IST. The weight reduction in successive days was considered as an amount of water lost *via* transpiration, and it was replenished to maintain the initial weight throughout the experiment period for well-watered (WW) pots, whereas water-stressed (WS) pots were made to mimic progressive soil drying conditions by gradually reducing the amount of water needed to replenish.

#### Normalized transpiration rate (NTR) and fraction of transpirable soil water (FTSW)

The point at which the transpiration rate of WS plant was 10% less than WW plants the experiment was terminated and the plant samples were collected for biomass calculation. As at this point the theoretical NTR value will be 0.1 (Sinclair & Ludlow, 1986), and the plant no longer sustains the stress situation. During the soil drying process, NTR was often expressed as a function of FTSW. To calculate FTSW, total transpirable water was calculated as a difference between initial pot weight and final pot weight *i.e*., where NTR is less than 10% or value 0.1, then daily FTSW was calculated as an amount of soil water remaining to the total transpirable soil water. Once experiment was terminated, the data pertaining to transpiration rates were normalized to facilitate the comparison. First normalization was done to avoid the environmental influence by calculating the daily transpiration ratio (TR). TR of each WS plant divided by mean TR of all WW plants of the corresponding genotype. Second normalization aims at avoiding differences due to plant size, calculated by dividing each TR value with an average TR value of the first 3 days when there was no water limitation. Detailed description on importance and application of NTR-FTSW was previously mentioned by various authors (Sinclair & Ludlow, 1986; Devi et al., 2009).

Transpiration efficiency (TE) was calculated as the ratio of biomass increase during the experiment period over the total water transpired.

TE = (Final biomass of plant – Biomass at beginning of experiment)/Total water transpired.

#### Physiological traits measured under pot and field conditions

**Figure 1:**
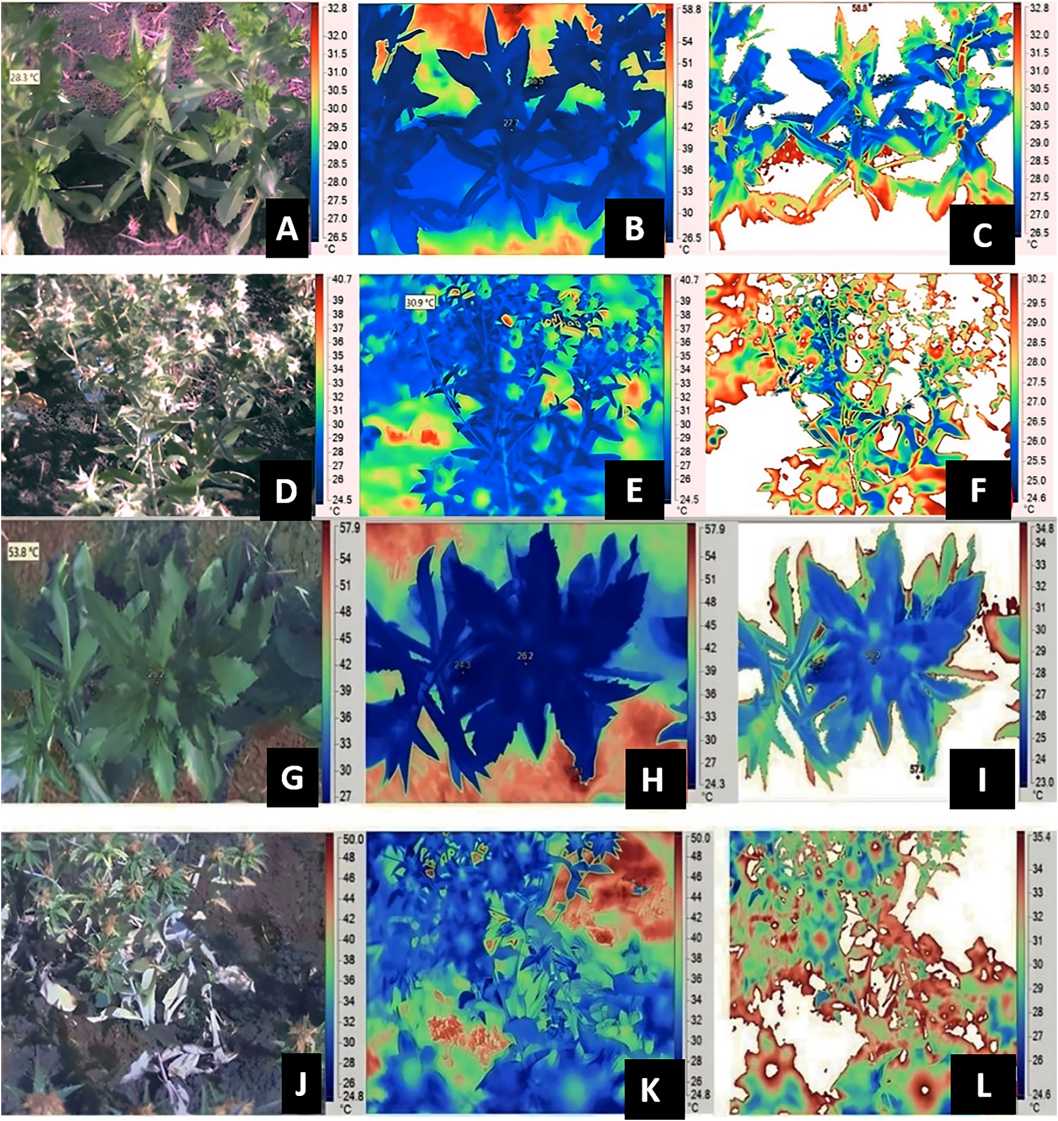
Estimation of canopy temperatures using IR thermography and image analysis in safflower genotype A-1. At vegetative (A and G) visual image (B and H) IR image (C and I) processed image; and reproductive stages (D and J) visual image (E and K) IR image (F and L) processed image under residual moisture conditions from replicates of thrice.

Data pertaining to SPAD chlorophyll metre, Infra-Red (IR) Camera, Infra Red Gas Analyzer (IRGA) were measured at both vegetative and flowering stage of respective genotypes, whereas RWC was measured only at flowering stage. SPAD-502 (Soil Plant Analytical Development; Konica, Ramsey, NJ, USA) metre was used for measuring the relative chlorophyll content of leaves. A third/fifth leaf from top of the plant (overall five samples) per plant were selected to record SPAD chlorophyll metre readings. The five readings from five individual leaves were pooled to get an average value, five plant data were recorded from each genotype for each replication. The gas-exchange measurements such as net photosynthesis, transpiration rate and stomatal conductance were recorded by using IRGA. Index leaf from tagged plants were used to record gas exchange parameters (IRGA; Model: LICOR 6400). The images were captured at vegetative and flowering stage, plant height corresponding to flowering stage was mentioned in Table S2. The thermal images of the plant canopies were captured using an infrared camera, the IR FLEXCAM (Infrared Solutions, Inc., Plymouth, MN, USA) with a sensor size of 160 × 120 pixels, sensitivity of 0.09 °C, accuracy of 2% and emissivity (ε) of 0.95. The images were taken from the north to prevent shading of the target region, which was approximately 30 cm × 20 cm at one of the middle rows of each plot (Kashiwagi et al., 2008). After removing the emissions from the soil (background), the image processing and estimation of canopy temperatures were done using the software SmartView 2.1.0.10 (Fluke Thermography, Everett, WA, USA) (Zaman-Allah, Jenkinson & Vadez, 2011a; Sivasakthi et al., 2017). The observations were with the camera attached to a shoulder at a. approximate height of 1.0 m. At the crop canopy level, a temperature and relative humidity recorder (Gemini Tiny Tag Ultra 2 TGU-4500 data logger, Chichester, UK) was used to measure the ambient temperature. The gas-exchange traits, SPAD chlorophyll meter readings and canopy temperature were captured during the early hours of 9:00 to 11:00 AM. CTD was calculated as the difference between the ambient and leaf temperature, *i.e*., CTD = Tambient – Tcanopy. Figure 1, provides insights into image capture and process after removal of background noisy data for genotypes A1 and GMU 2644 respectively. Relative leaf water content was measured based on the method described by Turner (1981).

### Experiment 2: Evaluation of safflower genotypes for CTD and yield traits in field under residual moisture conditions

The same set of 12 genotypes were further evaluated under field conditions for two years by growing them under residual moisture conditions using the recommended packages of practices given by ICAR-IIOR (Alivelu et al., 2014). Residual moisture conditions impart moderate to severe stress scenarios during the later stages of crop growth. Data pertaining to soil moisture levels, generated by gravimetric method revealed a reduction of 30.43 per cent from 10 DAS (vegetative) to 105 DAS (seed filling) at 15 to 45 cm depth, whereas it was 38 per cent at 45 to 60 cm. During both the seasons, the crops were sown by following a spacing of 45 × 20 cm, where only one sprinkler irrigation was given at sowing time, accounting for around 30 mm of water received. The experiments were conducted at ICAR-IIOR Research farm at ICRISAT, Patancheru (17.51°N, 78.27°E and 545 m above MSL). Information pertaining to prevailing climatic conditions in the experimental plot during experimentation were given in Fig. S1. The genotypes were raised in black soil (order: Vertisol) following randomized block design in an average plot size of 20 sq m accounting for an approximate 150 healthy plants. Observations of CTD, SPAD chlorophyll meter and gas exchange parameters were recorded similarly to the pot experiment at vegetative and reproductive stages in three replications. Morphological traits such as total biomass, leaf area index and yield traits were recorded at the time of harvest. Plant height was measured during flowering stage from tip to base with the help of 1 m scale from five tagged plants in each replication avoiding border rows. As for total dry matter the plants samples were harvested from base and samples were sundried for two days later oven dried at 72 °C till constant weight was attained. Leaf area index was measured from individual plots in replication with an AccuPAR LP-80 Ceptometer. Seeds from all the capitulum were pooled and calculated the total yield per plant. Harvest index (HI) = Economic yield/Biological yield was calculated at the end of experiments and the data recorded are given in Table S3.

### Statistical analysis

The experiments were carried out at two factors, genotype and progressive soil drying. In both the experiments, the data generated from both the years at respective vegetative, flowering and maturity stages were pooled before statistical analysis. To obtain replication mean, data sets generated for various traits were averaged. The mean data thus generated for various traits were compared at 1 per cent significance level using the least significance difference test. Further the mean data sets were analysed using appropriate statistical tools *viz*., FTSW-NTR threshold graphs using non-linear regression analysis, ANOVA was conducted for traits recorded from pot experiments *i.e*., TE (WW), TE (WS) and TDM (WS) to understand the existing variance between and within the genotypes under WW and WS conditions, comparison of TE (WW) and TE (WS) means using Tukey’s honestly significant difference test, trait associations among various traits measured under pot and field experiments, Pearson correlation analysis to understand the linear relations among the variables and PCA to reduce the size of the data sets generated and create a smaller set of new variables (principal components).

FTSW-NTR plots were generated for each genotype using individual replicated data on daily basis. GraphPad Prism 2.0 version 6 (GraphPad Software Inc., San Diego, CA, USA) was employed for construction of graphs and non-linear regression analysis to be in line with the exponential model given by Muchow & Sinclair (1991). R square values which quantify the goodness were generated using GraphPad Prism 2.0 version 6, which make use of sum of the squares of the distances of the points from the best-fit curve determined by nonlinear regression. The relationship among various morpho-physiological and yield traits were studied using linear regression analysis. Pearson correlation table was constructed to understand strong correlation and consistency among the traits using “corrplot” packages.

PCA for phenotypic traits recorded under residual moisture conditions was done using “FactomineR” (Husson, Josse & Pages, 2010), “factoextra” and “ggfortify” packages (R Development Core Team, 2008) under R software version 4.1.2 (R studio). Eighteen traits were selected for PCA analysis, involving calculating the covariance matrix, eigenvectors and eigenvalues of the covariance matrix. Principal components whose eigen values more than one were selected and PCA scores generated. The number of principal components to be retained in the analysis is often determined based on the amount of variance explained, with a threshold of 80% or greater commonly used. The biplot was generated by using the ‘FactoMineR’ (factor analysis and data mining) with function ‘biplot’.

## Results

### Experiment I: threshold levels of genotypes for NTR at FTSW

**Figure 2:**
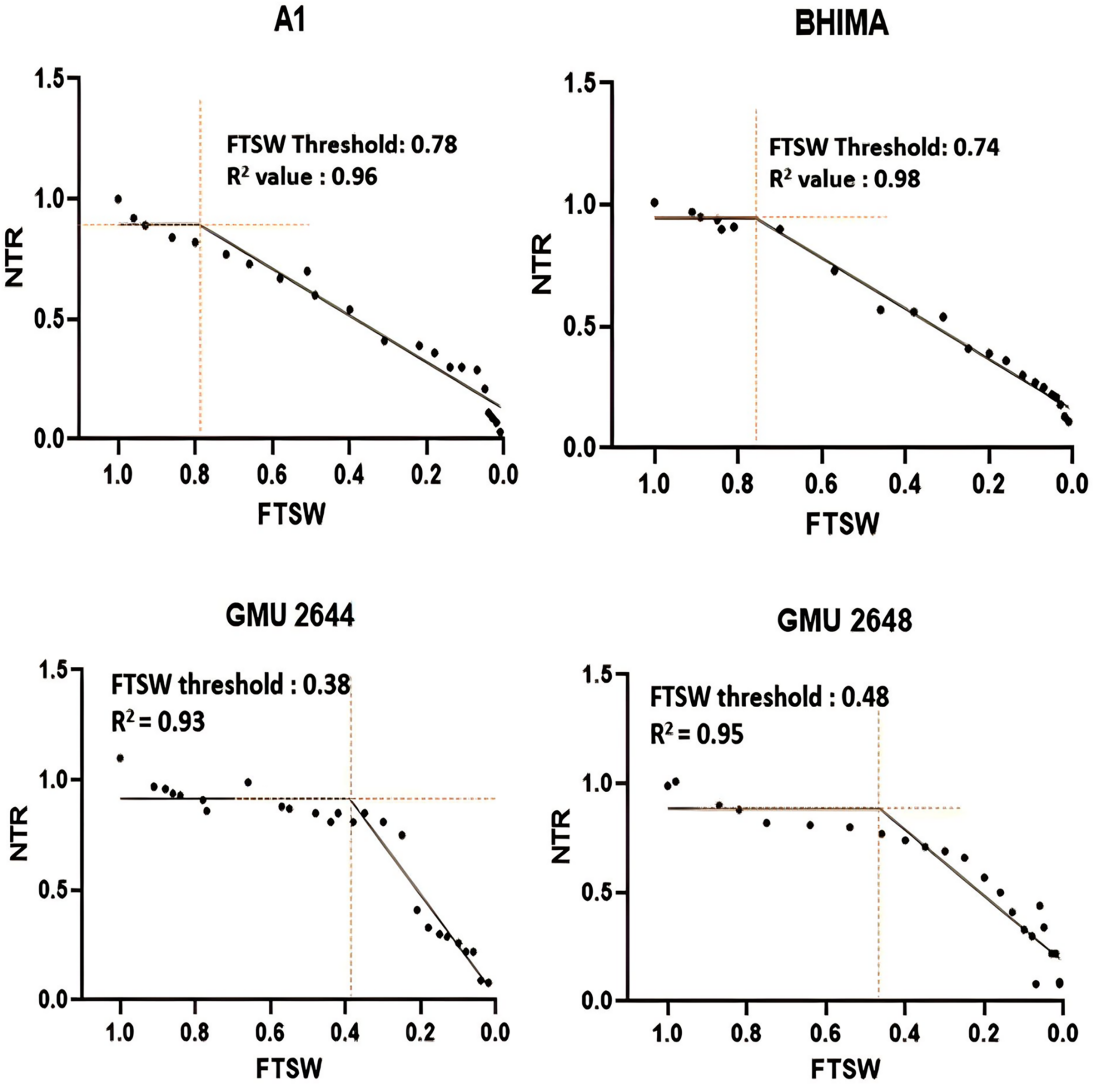
Variation in threshold levels in safflower genotypes for normalized transpiration rate (NTR) against fraction of transpirable soil water (FTSW). The fitted with threshold values were derived using non-linear regression exponential model. Data generated from five replication encompassing 10 plants each was used.

**Figure 3:**
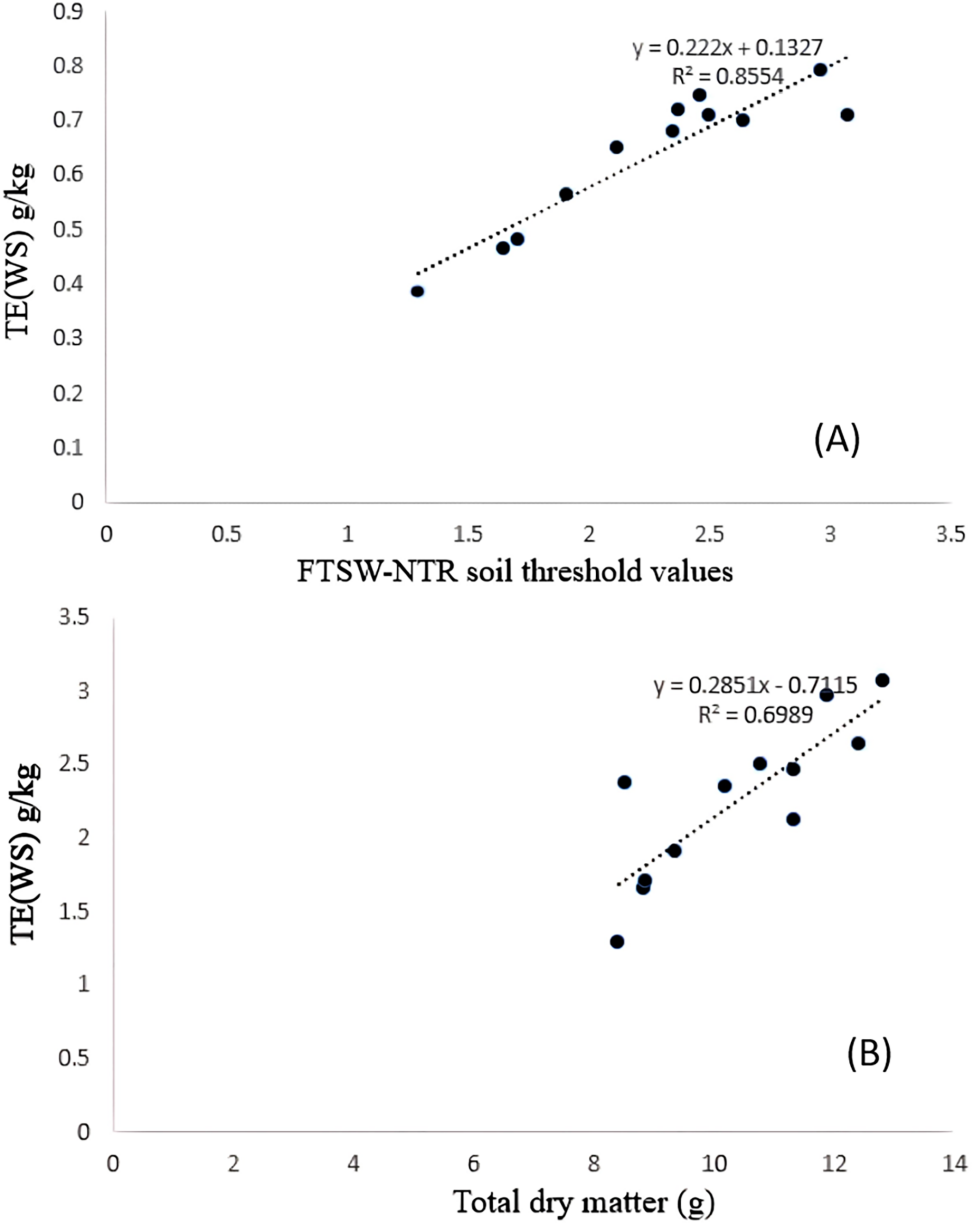
Relationship between (A) TE and FTSW-NTR & (B) TE and TDM under residual moisture stress conditions in safflower genotypes. Data generated from five replication encompassing 10 plants each were used.

Significant differences were noticed from pot experiments at genotype and treatment level for FTSW threshold values. Threshold values are the soil moisture points beyond which normal transpiration of the plant gets affected. Genotypes exhibited wide variation for FTSW – NTR threshold values. At a FTSW threshold of 0.79 (R^2^ = 0.99), genotype A-1 exhibited an early decline in NTR, followed by Bhima (0.74, R^2^ = 0.98), CO-1 (0.71, R^2^ = 0.91), GMU-2347 (0.71, R^2^ = 0.92), and EC-523368-2 (0.70, R^2^ = 0.99). Stress scoring that was done before the termination of experiments revealed a slow wilting followed by delayed senescence in genotypes CO-1, EC-523368-2, and NARI 6, which was attributed to their high soil moisture threshold. Furthermore, the adaptation governed by these genotypes was shown in terms of increased transpiration efficiency (TE) under stress, whereas genotypes (GMU-2644 (0.384, R^2^ = 0.93), followed by GMU-2648 (0.46, R^2^ = 0.90) and GMU 3438 (0.48, R^2^ = 0.95)) that recorded the lowest threshold value were often prone to moisture stress during later stages of growth (Fig. 2). There also existed a positive correlation between the FTSW threshold values and the TE of the genotypes under stress. FTSW as independent variable plotted against TE a dependent variable using linear regression analysis represented in following Eq. (1) (Fig. 3).

(1) $$\rm {y = 0.222x + 0.1327\, (adjusted\, R^2\, value\, 0.85) }$$

### Transpiration efficiency (TE) among the genotypes

TE varied significantly among genotypes under WW and WS conditions. Genotype CO-1 (4.88 g kg^−1^) recorded a high TE under WW, whereas GMU-2644 (2.56 g kg^−1^) recorded the least, 1.8 folds less than the highest value. A substantial reduction in mean TE was observed under WS conditions; genotypes GMU-2347 recorded the highest TE (2.72 g kg^−1^), which was on par with A-1 (2.66 g kg^−1^), EC-523368-2 (2.64 g kg^−1^) and CO-1 (2.496 g kg^−1^) with 0.23 CD. Similarly, the least TE was noticed on GMU-2644 (1.29 g kg^−1^), followed by GMU-2648 (1.65 g kg^−1^), GMU-3438 (1.71 g kg^−1^) and GMU-3266 (1.91 g kg^−1^) (Table 1). There was a significant positive relationship found in the amount of dry matter generated (above ground mass) and TE under WS conditions given in Eq. (2) (Fig. 3). ANOVA to understand the variance between and within the genotypes was given in Table S4.

**Table 1:** FTSW-NTR threshold values for selected genotypes and their TE under residual moisture stress conditions.

	FTSW-NTR	R^2^	TE (WS)	TE (WW)
EC-523368-2	0.7	0.99	2.64 bc	4.31 ab
A1	0.79	0.96	2.96 ab	4.82 a
BHIMA	0.74	0.98	2.46 cd	4.45 ab
CO-1	0.71	0.91	2.49 cd	4.88 a
GMU 2347	0.71	0.92	3.07 a	4.3 ab
GMU 2644	0.38	0.93	1.29 g	2.56 d
GMU 2648	0.46	0.9	1.65 fg	2.9 d
GMU 3266	0.56	0.95	1.91 ef	3.23 cd
GMU 3438	0.48	0.95	1.71 f	2.81 d
ISF 764	0.68	0.97	2.30 cd	3.95 b
NARI 6	0.72	0.95	2.37 cd	3.89 bc
PBNS 12	0.65	0.90	2.12 de	3.81 de
Range	0.38–0.79	0.90–0.99	1.29–3.07	2.56–4.88
Average	0.63	0.94	2.25	3.83
SD	0.13	0.03	0.54	0.79
SE	0.04	0.01	0.15	0.23
CV	0.21	0.03	0.24	0.21

**Note:**

Mean data of years 2020–21, 2021–22. Means within columns with the same letter(s) are not significantly different at 0.05 level of probability using Tukey’s honestly significant difference test.

(2) $$\rm {Y = 0.2851x - 0.7115\, (adjusted\, R^2\, value\, 0.698)}$$

### Trait association

Among the various traits recorded, results from linear regression analysis revealed that seed yield under residual soil moisture was significantly associated with harvest index (R^2^ = 0.39, *p* < 0.01) and weight of primary capitulum (R^2^ = 0.19, *p* < 0.01). Similarly, TE under WS was significantly associated with, total dry matter under stress (R^2^ = 0.69, *p* < 0.01), SPAD-chlorophyll meter reading (R^2^ = 0.26, *p* < 0.01), canopy temperature depression (vegetative stage) (R^2^ = 0.27, *p* < 0.01), canopy temperature depression (flowering) (R^2^ = 0.67, *p* < 0.01), leaf area index (R^2^ = 0.39, *p* < 0.01), assimilation rate (R^2^ = 0.15, *p* < 0.05) and relative water content (R^2^ = 0.67, *p* < 0.01) (Table 2).

**Table 2:** Regression analysis between seed yield and TE; TE and morpho-physiological traits of safflower genotypes under residual moisture conditions.

	Trait	R^2^ square value	LoS
Seed Yield *vs*	Harvest index	0.39*	*p* ≤ 0.05
	Weight of primary capitulum	0.2*	*p* ≤ 0.05
	Number of primary capitulum	0.2*	*p* ≤ 0.05
TE (WS) *vs*	Total dry matter	0.71*	*p* ≤ 0.05
	SPAD-Chlorophyll meter reading	0.26*	*p* ≤ 0.05
	Canopy temperature depression (Vegetative stage)	0.27*	*p* ≤ 0.05
	Canopy temperature depression (Flowering stage)	0.67*	*p* ≤ 0.05
	Leaf area index	0.39*	*p* ≤ 0.05
	Assimilation rate	0.15**	*p* ≤ 0.01
	Relative water content	0.67*	*p* ≤ 0.05

**Notes:**

* *p* ≤ 0.05.

** *p* ≤ 0.01.

Los, level of significance.

### Pearson correlation analysis

**Figure 4:**
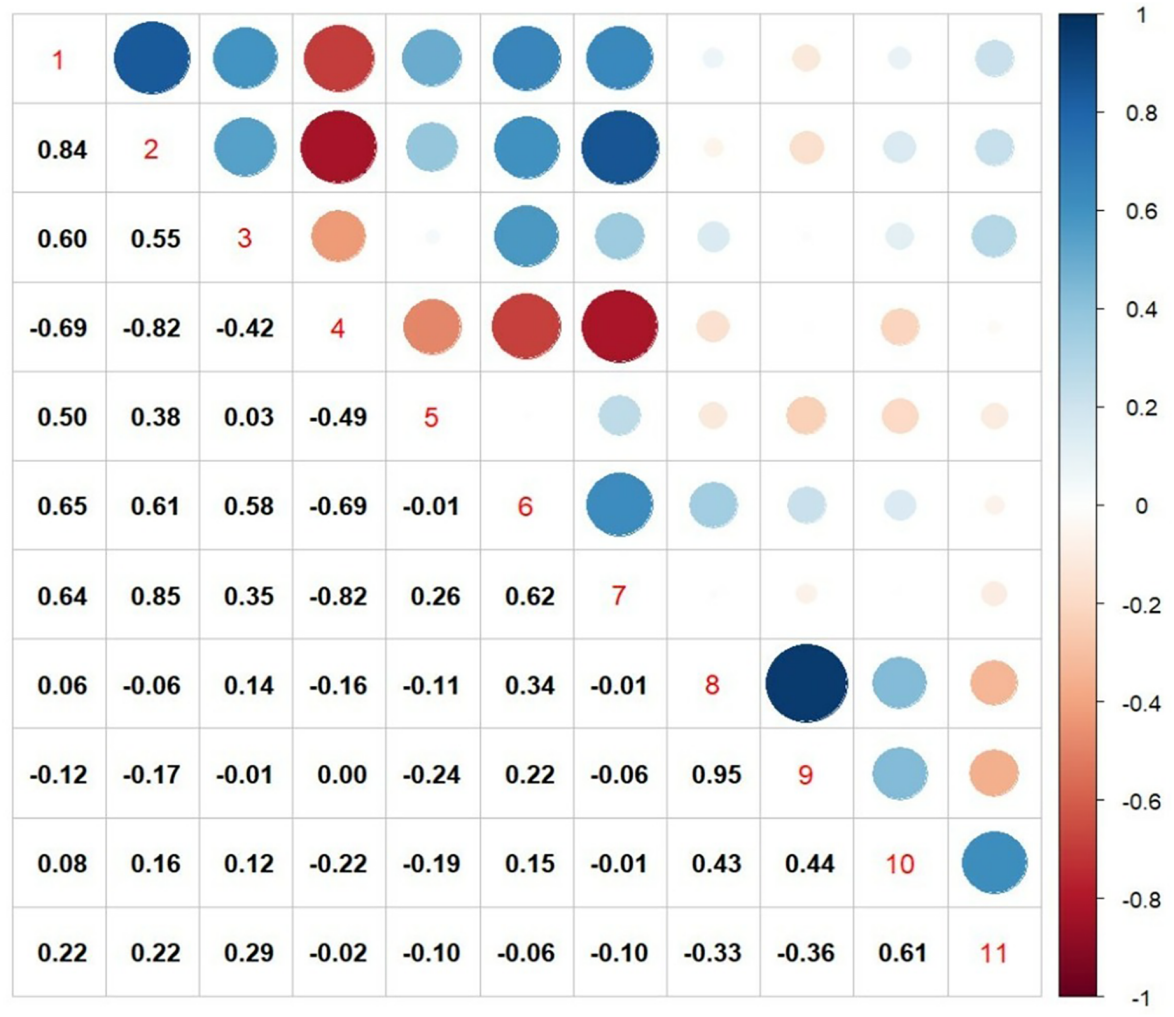
Pearson correlation matrix among various physiological traits with seed yield and harvest index. 1. TDM (WS), total dry matter under water stress; 2. TE (WS), transpiration efficiency under water stress; 3. CTD (V), canopy temperature depression at vegetative stage; 4. CTD (F), canopy temperature depression at flowering stage; 5. SCMR@60DAS, SPAD-chlorophyll meter readings at 60DAS; 6. LAI, leaf area index; 7. RWC, relative water content; 8. NPC, number of primary capitulum; 9. WPC, weight of primary capitulum; 10. seed yield; 11. HI, harvest index.

From both the experiments, the traits that were showing significant association with seed yield, were further subjected to correlation analysis and the results are given in Fig. 4. There was a positive correlation between transpiration efficiency under water stressed condition measured from pot experiment with, TDM under water stress (R = 0.83), CTD at vegetative stage (R = 0.54), SCMR values at flowering stage (R = 0.50), leaf area index (R = 0.65) and relative water content (R = 0.64), whereas a strong negative correlation was noticed from CTD at flowering stage (R = 0.69) under field conditions. Among various traits measured seed yield under field experiments exhibited a weak positive correlation TE under water stress (R = 0.15), number (R = 0.43) as well as weight of primary capitulum (R = 0.43) and strong positive correlation with harvest index (R = 0.61).

### Experiment II: genotypic differences in morpho-physiological and yield traits

From field experiments generated data, a significant difference was noticed among the genotypes for various morpho-physiological traits recorded under residual moisture conditions. NARI-6 (91.1 ± 2.1 cm) recorded the highest plant height on par with CO-1 (87.4 ± 4.0 cm), which is 1.6 folds higher than EC-523368-2 with least plant height (56.4 ± 2.137 cm). The leaf area index (LAI), trait that is proportional to transpiration and was measured at 50% flowering of genotypes. ISF-764 (1.13 ± 0.05), Bhima (1.1 ± 0.04) and GMU-2347 (1.1 ± 0.02) recorded the highest LAI under field conditions and were on par, followed by EC-523368-2 (1.02 ± 0.02) and A-1 (1.02 ± 0.02), whereas GMU-3438 (0.69 ± 0.03) and GMU-2648 (0.737 ± 0.02) recorded the least. In addition, genotypes that recorded the highest TE in pot experiments also recorded higher SCMR values in the field. The genotypes that differed significantly for SCMR values (*p* < 0.01) were PBNS-12 (55.45 ± 2.56), EC-523368-2 (52.37 ± 1.51), GMU-3266 (37.92 ± 1.31), and GMU-2644 (45.12 ± 1.04). Supporting the observations made from the pot experiment, genotypes A-1 (92.3 ± 2.13%), EC-523368-2 (87.3 ± 2.52%), Bhima (86.5 ± 2.99%) and GMU-2347 (84.7 ± 1.46%) had a relatively higher RWC, whereas genotype GMU-2648 (71.3 ± 2.05%) recorded a relatively lesser RWC, which was on par with GMU-3438 (72.4 ± 2.92%) and GMU-3266 (73.7 ± 2.55%) (Tables S2 and S3).

### Canopy temperature and gas exchange parameters

Exception to previous results, EC-523368-2 measured high transpiration rate (3.44 ± 0.09 m mol H_2_O m^−2^ s^−1^), high stomatal conductance (109 ± 3.147 m mol H_2_O m^−2^ s^−1^) on par with ISF-764 (108 ± 4.98 m mol H_2_O m^−2^ s^−1^), whereas net photosynthetic rate was higher in A-1 (29.5 ± 0.681µ CO_2_ m^−2^ s^−2^) on par with NARI 6 (27.9 ± 0.644 µ CO_2_ m^−2^ s^−2^) and at least by GMU-3266 (16.9 ± 0.58 (µ mol CO_2_ m^−2^ s^−1^)). Bhima recorded the highest leaf temperature (34.98 ± 0.66 °C), whereas the least was recorded from A1 and NARI 6 (29.76 ± 0.41 °C). Net photosynthetic rate recorded under field conditions is positively correlated with TE (WS) from pot conditions (R^2^ = 0.15, *p* < 0.05), whereas leaf temperature is related to seed yield under residual moisture (R^2^ = 0.19, *p* < 0.01) (Table S3).

### Seed yield and it’s components

The genotypes performed extremely well under well water conditions (Manikanta et al., 2023), whereas under residual soil moisture (RSM) conditions a reduction in yield ranging from 22 per cent to 42 per cent was noticed emphasizing the intensity of residual soil moisture stress the crop had undergone. Though genotypes NARI-6 and EC-523368-2 recorded high TE (correlated *via* SCMR values) and delayed senescence in field, both the genotypes recorded the least seed yield per plant. NARI 6 (8.50 ± 0.246 g) and EC-523368-2 (8.82 ± 0.204 g), whereas genotypes ISF-764 (21.694 ± 0.50 g) and A-1 (18.64 ± 0.7 g) recorded the highest seed yield under residual moisture stress (*p* < 0.01). Days to 50 per cent flowering showed no significant variation among the genotypes (*p* > 0.05). The Harvest index (HI) significantly varied (*p* < 0.01) among the genotypes, with the highest in ISF-764 (0.369 ± 0.0005), which was on par with A-1 (0.366 ± 0.00031) (Table S2).

### Analysis of principal components

**Figure 5:**
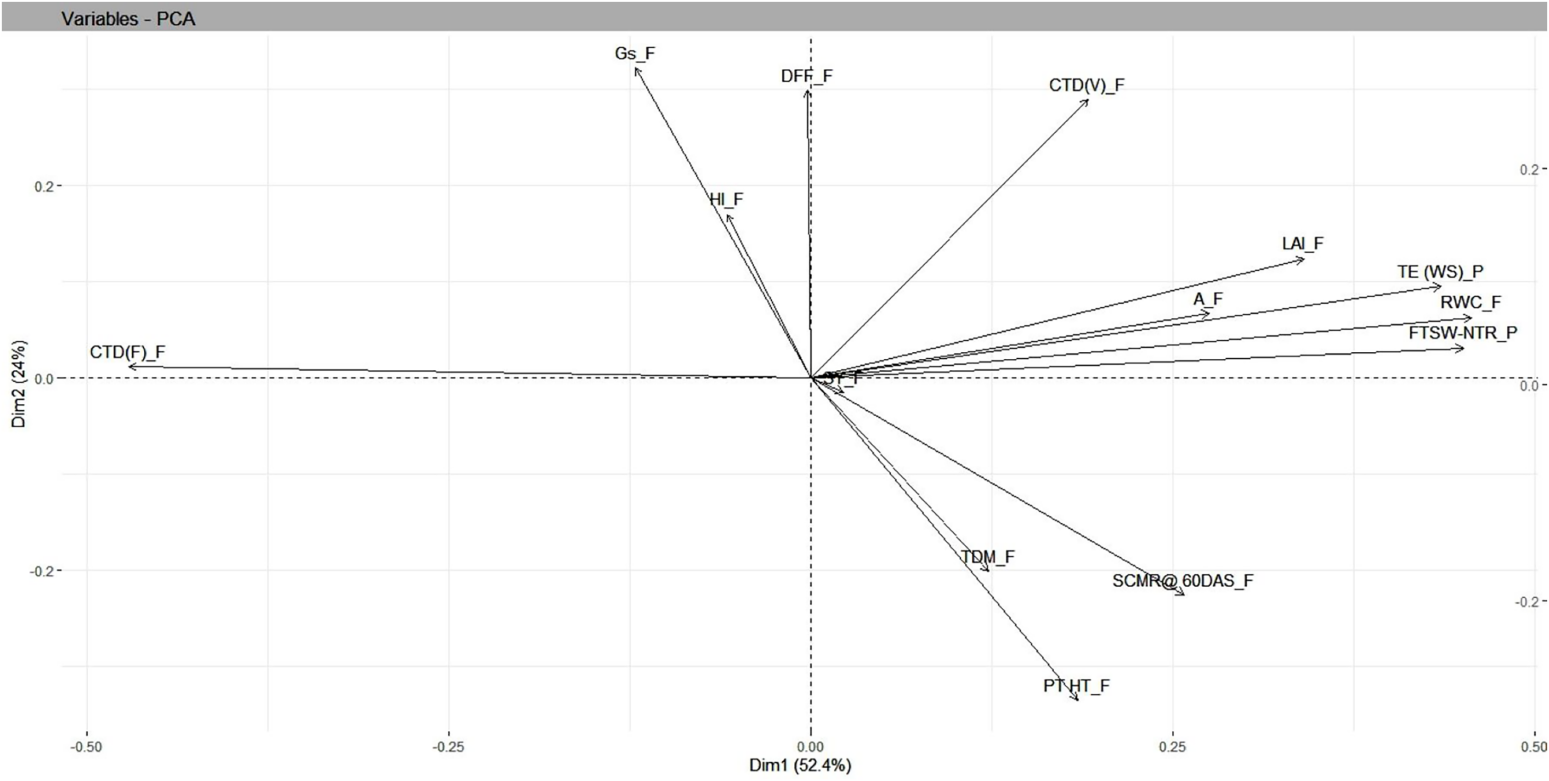
Genotype-by-trait biplot analysis of safflower genotypes between PCs 1 and 2, shows the contribution of various traits under residual moisture conditions. FTSW-NTR_P, FTSW-NTR threshold value; TE_WS_P, transpiration efficiency under water stress; CTD_V, canopy temperature depression at vegetative stage; CTD_F, canopy temperature depression at flowering; SCMR_F, SPAD chlorophyll meter reading; DFL, days to 50 per cent flowering; LAI, leaf area index; RWC, relative water content; TDM_F, total dry matter at field conditions; SY, seed yield; HI, harvest index; A_F, assimilation rate in field; Gs_F, stomatal conductance at flowering. SD, standard.

Under residual soil moisture conditions, PCA resulted in four independent components whose SD values were more than 1, accounting for a cumulative variance of 88 percent and with SD values greater than 1.13. The maximum variation in PC1 was accounted for by FTSW-NTR threshold values (0.42), TE under WS (0.37), and RWC under field (0.36), while the least variation was accounted for days to 50% flowering (−0.15). In PC2, TE under WS (0.33), days to 50% flowering (0.32), and harvest index (−0.38) contributed the most variation, with capitulum weight under field (−0.43), total dry matter under field (−0.38), and seed yield under field (−0.38) contributing the least. Findings from biplot revealed a closer association, *i.e*., a smaller angle between the vectors between seed yield and yield attributing traits in safflower that included plant height and total dry matter, whereas physiological traits such as RWC, CTD at vegetative stage, FTSW-NTR thresholds, and morphological traits such as leaf area index were in close relationship with TE under water stress in pot conditions (Table 3 and Fig. 5).

**Table 3:** Eigen values, proportion of variation and component for seed yield components in safflower genotypes express the non-rotated loadings under residual moisture conditions.

Loadings:	PC1	PC2	PC3	PC4	PC5	PC6	PC7	PC8	PC9	PC10
FTSW.NTR_P	0.42	0.23	0.00	0.00	0.25	0.23	0.00	0.49	0.58	0.00
TE_WS_P	0.37	0.33	0.00	0.14	0.11	0.00	0.31	0.28	−0.45	0.00
CTD_V_F	0.22	0.28	−0.19	−0.22	−0.69	0.48	0.10	−0.15	0.00	−0.19
SCMR_F	0.00	0.16	0.22	0.71	−0.16	0.19	−0.49	−0.13	−0.14	0.26
DFL_F	−0.15	0.32	0.00	−0.54	0.34	0.36	−0.44	0.00	−0.23	0.31
LAI_F	0.37	0.15	0.00	−0.29	−0.33	−0.67	−0.42	0.00	0.00	0.00
RWC_F	0.36	0.28	0.20	0.00	0.34	−0.11	0.22	−0.75	0.00	0.00
TDM_F	0.33	−0.38	0.20	0.00	0.00	0.00	0.00	0.19	−0.58	0.00
CW_F	0.25	−0.43	0.17	−0.14	−0.19	0.17	0.21	−0.11	0.19	0.68
SY_F	0.26	−0.22	−0.54	0.00	0.15	0.00	−0.22	−0.12	0.00	0.00
HI_F	0.00	0.13	−0.70	0.13	0.00	−0.16	0.11	0.00	0.00	0.43
RSY_N_HI	0.32	−0.38	−0.14	0.00	0.15	0.18	−0.36	−0.13	0.00	−0.38
SD	1.99	1.85	1.36	1.18	0.85	0.55	0.47	0.32	0.18	0.13
PV	0.33	0.29	0.15	0.12	0.06	0.02	0.02	0.01	0.00	0.00
CPV	0.33	0.61	0.77	0.88	0.94	0.97	0.99	1.00	1.00	1.00

**Note:**

FTSW-NTR_P, FTSW-NTR threshold value; TE_WS_P, transpiration efficiency under water stress; CTD_V, canopy temperature at depression vegetative stage; SCMR_F, SPAD chlorophyll meter reading at flowering; DFL, days to 50 per cent flowering; LAI, leaf area index; RWC, relative water content; TDM, total dry matter; SY, seed yield; HI, harvest index; RSY_N_HI, residual seed yield devoid of harvest index effect; SD, standard deviations; PV, proportional variance; CPV, cumulative proportional variance; P, pot; F, field.

## Discussion

A significant difference was revealed for possible genotypic variation for transpiration efficiency under progressive soil drying conditions. Genotypes with higher threshold values for FTSW often restrict the amount of water loss *via* transpiration. FTSW threshold for genotype A-1: 0.79 (R^2^ = 0.99), followed by Bhima: 0.74, (R^2^ = 0.98), CO-1: 0.71 (R^2^ = 0.91), and GMU-2347: 0.71, (R^2^ = 0.92) had high threshold values as well as TE, strengthening the theoretical fact (Sinclair & Ludlow, 1986) (Table 1 and Fig. 2). Devi & Reddy (2020) from their experiments on maize genotypes revealed that at higher VPD (vapour pressure deficit) genotypes limited their transpiration rate exhibiting high FTSW threshold values. Sinclair et al. (2017) and Devi & Reddy (2020) postulated that limited transpiration trait expressed could contribute to early season moisture conservation and as an outcome, crop yields are improved under drought. The adaptation mechanism involved in such genotypes may be associated with the early stomatal closure under mid-day conditions (high vapour pressure deficit (VPD) conditions) where gaseous exchange becomes zero (Riar et al., 2018; Devi et al., 2009). Though the negative impact of stomatal closure will be evident by reduction in assimilation rate, it will be well compensated by the soil moisture conserved in the early stages of growth, by sustaining physiological processes during late stages of crop growth, thereby increasing the TE of the genotype. From the two trials, it is evident that some genotypes have the capacity to respond to soil moisture depletion and regulate their transpiration well ahead of actual hydraulic limitations in soil. Devi et al. (2009) reported similar results, where peanut genotype ICG 11376 recorded a threshold value of 0.71. Plants experience water stress situations when the amount of water transpired is not actively replenished. Two main forces that determine the water uptake in plants are hydrostatic forces created by transpiration and osmotic forces by root pressure (Aroca, Porcel & Ruiz-Lozano, 2012). From the study, it is evident that significant genetic variation exists among the genotypes for TE, under WS condition than under WW condition emphasizing the fact that progressive soil drying has a major impact on TE. A high TE may be the result of increased biomass with fixed water input or fixed biomass with a reduction in water input, or a combined effect of both (Xin, Aiken & Burke, 2009). The low TE of genotype GMU-2648 could be attributed to unregulated transpiration rates during early stages of crop growth, resulting less water use efficiency, whereas the high TE of genotypes CO-1, A-1, NARI-6, and EC-523368-2 could be due to “more” biomass for unit water consumed, which resulted in low water consumption. In line with the current study, a positive correlation of r = 0.74 between TE and total biomass among the genotypes was reported in sorghum (Xin, Aiken & Burke, 2009; Condon et al., 2004).

The positive relationship of TE with SCMR reading (R^2^ = 0.26, *p* = 0.01) and with canopy temperature depression (CTD) (R^2^ = 0.67, *p* = 0.01) (Table 2) measured at flowering stage suggested that SCMR and CTD could serve as surrogate traits for TE in safflower. Similar findings in peanut have been reported (Madhava et al., 2003; Sheshshayee et al., 2006; Devi et al., 2011; Belko et al., 2012).

Safflower grown under residual moisture stress is often prone to terminal drought. Current studies emphasis the same, a reduction of 38.3 per cent of soil moisture from 10 to 105 DAS certainly induced moisture stress in later stages of crop growth. The field experiments were conducted to confirm the genotypic variation in adaptation to moisture stress. A scarcity of water activates a wide range of response mechanisms at the physiological, biochemical, and molecular levels, ensuring a change in plant metabolic pathways (Koocheki, Rezvani & Fallahi, 2016). These alterations lead to physiological disorders, mainly a decrease in transpiration and photosynthetic rate, a reduction in chlorophyll content and the production of reactive oxygen species (ROS) (Ebrahimian et al., 2019). Among various morpho-physiological traits, plant height is often used as a selection criterion for genotypes in safflower (Nabloussi et al., 2008). The height of the plants ranged from 91.6 cm in NARI-6 to 56.4 cm in EC-523368-2, and the LAI ranged from 1.13 in ISF-764 to 0.69 in GMU-3438. The existence of large variation for these morphological traits is primarily due to genetic control, but environmental factors such as altitude, air and soil temperature, and soil moisture also influence phenotypic expression (La Bella et al., 2019). Our studies emphasized the possible roles of SCMR values as surrogate tool to measure TE in field conditions. Singh et al. (2014) and Darkwa et al. (2016) from their studies reported the same. Nigam & Aruna (2008) reported that the peanut genotype ICGV 99029 with a higher SCMR value and low SLA, which is often considered as the parental line for water use efficiency trait, making it a reliable tool to correlate TE under field conditions (Tables S2 and S3).

Genotypes with higher SCMR values, CO-1 (61.5 ± 2.85), EC-523368-2 (52.37 ± 1.51) also exhibited a delay in senescence. These findings are in line with Kassahun et al. (2010), who demonstrated that delayed leaf senescence often confers tolerance to post flowering moisture stress in sorghum. RWC is often used as a screening tool for drought tolerance, as water content and water potential are considered physiologically appropriate indicators of drought effects (Mohammadi et al., 2016). Tolerant genotypes often maintain higher values of RWC in stress, as evident in the present study by genotypes A1 (92.3 ± 2.13), EC-523368-2 (87.3 ± 2.52), Bhima (86.5 ± 2.99) and GMU 2347 (84.7 ± 1.46). Stomatal-driven transpiration regulation is a common mechanism to tolerate water stress (Shardendu, Singh & Reddy, 2011). Our current study also emphasized the importance of mid-day stomatal closure in achieving higher TE. In the present study, except EC-523368-2, all other genotypes that were found to perform better for various physiological traits exhibited lower stomatal conductance and transpiration rate. Adaptation to the adverse effects of moisture stress with limited gaseous exchange was due to stomatal closure, under such circumstances genotypes that maintain higher photosynthetic rates are often desirable. Though closure of stomata limits the gaseous exchange, availability of soil moisture for longer periods of crop growth compensates the negative impacts of stomatal closure and enhance the duration for photosynthesis. A-1 (29.5 ± 0.681) on par with NARI 6 (27.9 ± 0.644) even with limited stomatal conductance and transpiration rate, maintained better photosynthesis strengthening the theoretical fact.

The major expected outcome of any breeding programmes designed is to obtain a high yield coupled with quality. A significant positive relation was reported between seed yield and plant height, number of heads, and days to flowering. In this study, ISF 764 and A1 recorded the highest seed yield and high number as well as weight of capitulum. Camas, Cirak & Esendal (2007), Golkar, Arzani & Rezaei (2012), and Ali et al. (2020) emphasised the importance of capitulum number and weight per plant in safflower. These genotypes also exhibited better TE under pot conditions owing to their tolerance. The harvest index, which measures the reproductive efficiency of the crop, was found to be highest in genotypes that tolerate stress situations (Champolivier & Merrien, 1996; Unkovich, Baldock & Forbes, 2010). In the present study, genotypes ISF 764 and A1 that are better performing for various morpho-physiological traits recorded the highest HI.

## Conclusion

To conclude, the residual moisture stress is frequent among post-rainy season crops. A genotype with the ability to regulate water uptake and utilisation during early stages of growth with maintaining higher FTSW-NTR thresholds confers tolerance to moisture stress. Among the genotypes, A-1, Bhima, ISF-764, and GMU-2347 were reported higher FTSW-NTR thresholds, limiting their transpiration rate while the soil is relatively moist. This indicates that these genotypes adapt a moisture conservative approach in anticipation of stress in later stages of crop growth. The trait association revealed the presence of a positive relation between SCMR, CTD with TE often making them as surrogate tool to measure TE under field conditions. Furthermore, these traits could be employed as a screening tool for safflower adaption to WS conditions. Overall, the genotypes A-1, Bhima, GMU-2347 and ISF-764, had soil moisture conservation mechanisms during the vegetative stage, which facilitate them to adapt residual soil moisture. In addition, these genotypes could be used in safflower breeding programmes to develop cultivars for water-limited environments.

## Supplemental Information

10.7717/peerj.15928/supp-1 Supplemental Information 1 Climatic conditions from experimental plots during crop period *i.e*., post *rainy* 2020–21 & 2021–22.

10.7717/peerj.15928/supp-2 Supplemental Information 2 List of genotypes selected for study.

10.7717/peerj.15928/supp-3 Supplemental Information 3 Yield and yield attributes data of 12 safflower genotypes recorded under RSM conditions (2020–21, 22). Biomass, total dry matter at maturity stage; NPC, number of primary capitulum; NSC, number of secondary capitulum; WM, weight of main capitulum; WPC, weight of primary capitulum; SY, seed yield per plant; HI, harvest index.

10.7717/peerj.15928/supp-4 Supplemental Information 4 Physiological and phenological data of 12 safflower genotypes recorded under RSM conditions (2020–21, 22). SCMR, SPAD chlorophyll meter readings; RWC, relative water content; LAI, leaf area index. STDEV, standard deviation; SE, standard error; CV, coefficient of variation.

10.7717/peerj.15928/supp-5 Supplemental Information 5 ANOVA emphasizing variance between the genotypes and the variance within genotypes. SS, sum of squares; df, degrees of freedom; MS, mean sum of square; F, F value.

10.7717/peerj.15928/supp-6 Supplemental Information 6 Pooled Raw Data from pot and field experiments.

10.7717/peerj.15928/supp-7 Supplemental Information 7 Raw Data recorded and subsequently calculated FTSW-NTR data points throughout the experimental periods. Each data point represents data generated by pooling two seasons data, at five replications for each genotype.

## Additional Information and Declarations

### Data Availability

The following information was supplied regarding data availability: The raw data is available in the Supplemental Files. The genotypes selected for the studies and the pooled phenotypic data generated from all the 2 years experiments were presented in Tables S1–S3.

## References

[ref-1] Ali F, Yilmaz A, Chaudhary HJ, Nadeem MA, Rabbani MA, Arslan Y, Nawaz MA, Habyarimana E, Baloch FS (2020). Investigation of morphoagronomic performance and selection indices in the international safflower panel for breeding perspectives. Turkish Journal of Agriculture and Forestry.

[ref-2] Alivelu K, Padmavathi P, Srinivas PS, Prasad RD, Anjani G, Padmaiah M (2014). Safflower management practices.

[ref-3] Aroca R, Porcel R, Ruiz-Lozano JM (2012). Regulation of root water uptake under abiotic stress conditions. Journal of Experimental Botany.

[ref-4] Bagherzadi L, Sinclair TR, Zwieniecki M, Secchi F, Hoffmann W, Carter TE, Rufty TW (2017). Assessing water-related plant traits to explain slow-wilting in soybean PI 471938. Journal of Crop Improvement.

[ref-5] Belko N, Zaman-Allah M, Cisse N, Diop NN, Zombre G, Ehlers JD, Vadez V (2012). Lower soil moisture threshold for transpiration decline under water deficit correlates with lower canopy conductance and higher transpiration efficiency in drought-tolerant cowpea. Functional Plant Biology.

[ref-6] Camas N, Cirak C, Esendal E (2007). Seed yield, oil content and fatty acids composition of safflower (*Carthamus tinctorius* L.) grown in northern Turkey conditions. Anadolu Tarım Bilimleri Dergisi.

[ref-7] Champolivier L, Merrien A (1996). Effects of water stress applied at different growth stages to *Brassica napus* L. var. oleifera on yield, yield components and seed quality. European Journal of Agronomy.

[ref-8] Christy B, Tausz-Posch S, Tausz M, Richards R, Rebetzke G, Condon A, McLean T, Fitzgerald G, Bourgault M, O’Leary G (2018). Benefits of increasing transpiration efficiency in wheat under elevated CO_2_ for rainfed regions. Global Change Biology.

[ref-9] Condon AG, Richards RA, Rebetzke GJ, Farquhar GD (2004). Breeding for high water-use efficiency. Journal of Experimental Botany.

[ref-10] Darkwa K, Ambachew D, Mohammed H, Asfaw A, Blair MW (2016). Evaluation of common bean (*Phaseolus vulgaris* L.) genotypes for drought stress adaptation in Ethiopia. The Crop Journal.

[ref-11] Devi MJ, Bhatnagar-Mathur P, Sharma KK, Serraj R, Anwar SY, Vadez V (2011). Relationships between transpiration efficiency and its surrogate traits in the rd29A: DREB1A transgenic lines of groundnut. Journal of Agronomy and Crop Science.

[ref-12] Devi MJ, Reddy VR (2020). Stomatal closure response to soil drying at different vapor pressure deficit conditions in maize. Plant Physiology and Biochemistry.

[ref-13] Devi MJ, Sinclair TR, Vadez V, Krishnamurthy L (2009). Peanut genotypic variation in transpiration efficiency and decreased transpiration during progressive soil drying. Field Crops Research.

[ref-14] Ebrahimian E, Seyyedi SM, Bybordi A, Damalas CA (2019). Seed yield and oil quality of sunflower, safflower, and sesame under different levels of irrigation water availability. Agricultural Water Management.

[ref-15] Edwards CE, Ewers BE, Weinig C (2016). Genotypic variation in biomass allocation in response to field drought has a greater affect on yield than gas exchange or phenology. BMC Plant Biology.

[ref-16] Fahad S, Bajwa AA, Nazir U, Anjum SA, Farooq A, Zohaib A, Sadia S, Nasim W, Adkins S, Saud S, Ihsan MZ (2017). Crop production under drought and heat stress: plant responses and management options. Frontiers in Plant Science.

[ref-17] Faralli M, Williams KS, Han J, Corke FM, Doonan JH, Kettlewell PS (2019). Water-saving traits can protect wheat grain number under progressive soil drying at the meiotic stage: a phenotyping approach. Journal of Plant Growth Regulation.

[ref-18] Geetika G, Van Oosterom EJ, George-Jaeggli B, Mortlock MY, Deifel KS, McLean G, Hammer GL (2019). Genotypic variation in whole-plant transpiration efficiency in sorghum only partly aligns with variation in stomatal conductance. Functional Plant Biology.

[ref-19] Golkar P, Arzani A, Rezaei A (2012). Genetic analysis of agronomic traits in safflower (*Carthamus tinctorious* L.). Notulae Botanicae Horti Agrobotanici Cluj-Napoca.

[ref-20] Heinemann AB, Stone LF, Fageria NK (2011). Transpiration rate response to water deficit during vegetative and reproductive phases of upland rice cultivars. Scientia Agricola.

[ref-21] Husson F, Josse J, Pages J (2010). Principal component methods-hierarchical clustering-partitional clustering: why would we need to choose for visualizing data. Applied Mathematics Department.

[ref-22] Javed S, Ashraf MY, Mahmood S, Bukhari SA, Meraj M, Perveen A (2013). Comparative evaluation of biochemical changes in different safflower varieties (*Carthamus tinctorius* L.) under water deficit. Journal Food Process Technology.

[ref-23] Johnson RC, Bergman JW, Flynn CR (1999). Oil and meal characteristics of core and non-core safflower accessions from the USDA collection. Genetic Resources and Crop Evolution.

[ref-24] Kashiwagi J, Krishnamurthy L, Upadhyaya HD, Gaur PM (2008). Rapid screening technique for canopy temperature status and its relevance to drought tolerance improvement in chickpea. Journal of SAT Agricultural Research.

[ref-25] Kassahun B, Bidinger FR, Hash CT, Kuruvinashetti MS (2010). Stay-green expression in early generation sorghum [*Sorghum bicolor* (L.) Moench] QTL introgression lines. Euphytica.

[ref-26] Katerji N, Mastrorilli M, Rana G (2008). Water use efficiency of crops cultivated in the Mediterranean region: review and analysis. European Journal of Agronomy.

[ref-27] Khalili M, Alireza PA, Naghavi MR, Mohammad-Amini E (2014). Evaluation of drought tolerance in safflower genotypes based on drought tolerance indices. Notulae Botanicae Horti Agrobotanici Cluj-Napoca.

[ref-28] Kholová J, Hash CT, Kakkera A, Kočová M, Vadez V (2010). Constitutive water-conserving mechanisms are correlated with the terminal drought tolerance of pearl millet [*Pennisetum glaucum* (L.) R. Br.]. Journal of Experimental Botany.

[ref-29] Koocheki A, Rezvani MP, Fallahi HR (2016). Effects of planting dates, irrigation management and cover crops on growth and yield of saffron (*Crocus sativus* L.). Agroecology.

[ref-30] La Bella S, Tuttolomondo T, Lazzeri L, Matteo R, Leto C, Licata M (2019). An agronomic evaluation of new safflower (*Carthamus tinctorius* L.) germplasm for seed and oil yields under Mediterranean climate conditions. Agronomy.

[ref-31] Liu F, Andersen MN, Jacobsen SE, Jensen CR (2005). Stomatal control and water use efficiency of soybean (*Glycine max* L. Merr.) during progressive soil drying. Environmental and Experimental Botany.

[ref-32] Madhava HB, Sheshshayee MS, Shankar AG, Prasad TG, Udayakumar M (2003). Use of SPAD chlorophyll meter to assess transpiration efficiency of peanut. Breeding of drought resistant peanuts. ACIAR Proceedings.

[ref-33] Manikanta CLN, Ratnakumar P, Pandey BB, Guhey A, Prayaga L, Padmavathi P, Qureshi MAA, Mukta N, Kadirvel P (2023). Exploitation of the phenotypic diversity of safflower (*Carthamus tinctorius* L.) for deficit soil moisture stress tolerance.

[ref-34] Minhas PS, Rane J, Pasala RK (2017). Abiotic stress management for resilient agriculture.

[ref-35] Mohammadi M, Ghassemi-Golezani K, Zehtab-Salmasi S, Nasrollahzade S (2016). Assessment of some physiological traits in spring safflower (*Carthamus tinctorius* L.) cultivars under water stress. International Journal of Life Science.

[ref-36] Muchow RC, Sinclair TR (1991). Water deficit effects on maize yields modelled under current and ‘‘greenhouse’’ climates. Agronomy Journal.

[ref-37] Nabloussi A, El Fechtali M, Lyagoubi S, Knights S, Potter T (2008). Agronomic and technological evaluation of a world safflower collection in Moroccan conditions.

[ref-38] Nigam SN, Aruna R (2008). Stability of soil plant analytical development (SPAD) chlorophyll meter reading (SCMR) and specific leaf area (SLA) and their association across varying soil moisture stress conditions in groundnut (*Arachis hypogaea* L.). Euphytica.

[ref-39] Pandey BB, Ratnakumar P, Kiran BU, Dudhe MY, Lakshmi GS, Ramesh K, Guhey A (2021). Identifying traits associated with terminal drought tolerance in sesame (*Sesamum indicum* L.) genotypes. Frontiers in Plant Science.

[ref-40] R Development Core Team (2008). R: a language and environment for statistical computing. http://www.R-project.org.

[ref-41] Rahmani F, Sayfzadeh S, Jabbari H, Valadabadi SA, Hadidi Masouleh E (2019). Alleviation of drought stress effects on safflower yield by foliar application of zinc. International Journal of Plant Production.

[ref-42] Ratnakumar P, Pandey BB, Gandi SL, Kulasekaran R, Guhey A, Vishnuvardhan Reddy A (2021). An insight into the mechanisms of intermittent drought adaptation in sesame (*Sesamum indicum* L.): linking transpiration efficiency and root architecture to seed yield. Acta Physiologiae Plantarum.

[ref-43] Ratnakumar P, Vadez V (2011). Tolerant groundnut (*A. hypogaea* L.) genotypes to intermittent drought maintains high harvest index and has small leaf canopy under stress. Functional Plant Biology.

[ref-44] Ratnakumar P, Vadez V, Nigam SN, Krishnamurthy L (2009). Assessment of transpiration efficiency in peanut (*Arachis hypogaea* L.) under drought using a lysimetric system. Plant Biology.

[ref-45] Ray JD, Gesch RW, Sinclair TR, Hartwell Allen L (2002). The effect of vapor pressure deficit on maize transpiration response to a drying soil. Plant and Soil.

[ref-46] Riar MK, Cerezini P, Manandhar A, Sinclair TR, Li Z, Carter TE (2018). Expression of drought-tolerant N_2_ fixation in heterogeneous inbred families derived from PI471938 and Hutcheson Soybean. Crop Science.

[ref-47] Royo C, Villegas D, Del Moral LG, Elhani S, Aparicio N, Rharrabti Y, Araus JL (2002). Comparative performance of carbon isotope discrimination and canopy temperature depression as predictors of genotype differences in durum wheat yield in Spain. Australian Journal of Agricultural Research.

[ref-48] Shardendu K, Singh K, Reddy R (2011). Regulation of photosynthesis, fluorescence, stomatal conductance and water-use efficiency of cowpea (*Vigna unguiculata* [L.] Walp.) under drought. Journal of Photochemistry and Photobiology.

[ref-49] Sheshshayee MS, Bindumadhava H, Rachaputi NR, Prasad TG, Udayakumar M, Wright GC, Nigam SN (2006). Leaf chlorophyll concentration relates to transpiration efficiency in peanut. Annals of Applied Biology.

[ref-51] Sinclair TR, Ludlow MM (1986). Influence of soil water supply on the plant water balance of four tropical grain legumes. Functional Plant Biology.

[ref-62] Sinclair TR, Devi J, Shekoofa A, Choudhary S, Sadok W, Vadez V, Riar M, Rufty T (2017). Limited-transpiration response to high vapor pressure deficit in crop species. Plant Science.

[ref-52] Singh AL, Nakar RN, Chakraborty K, Kalariya KA (2014). Physiological efficiencies in mini-core peanut germplasm accessions during summer season. Photosynthetica.

[ref-53] Sivasakthi K, Marques E, Kalungwana N, Carrasquilla-Garcia N, Chang PL, Bergmann EM, Bueno E, Cordeiro M, Sani SGAS, Udupa SM, Rather IA, Rouf Mir R, Vadez V, Vandemark GJ, Gaur PM, Cook DR, Boesch C, von Wettberg EJB, Kholova J, Penmetsa RV (2019). Functional dissection of the chickpea (*Cicer arietinum* L.) stay-green phenotype associated with molecular variation at an ortholog of Mendel’s I gene for cotyledon color: implications for crop production and carotenoid biofortification. International Journal of Molecular Sciences.

[ref-54] Sivasakthi K, Tharanya M, Kholová J, Wangari Muriuki R, Thirunalasundari T, Vadez V (2017). Chickpea genotypes contrasting for vigor and canopy conductance also differ in their dependence on different water transport pathways. Frontiers in Plant Science.

[ref-55] Turner NC (1981). Techniques and experimental approaches for the measurement of plant water status. Plant and Soil.

[ref-56] Unkovich M, Baldock J, Forbes M (2010). Variability in harvest index of grain crops and potential significance for carbon accounting: examples from Australian agriculture. Advances in Agronomy.

[ref-57] Vadez V, Ratnakumar P (2016). High transpiration efficiency increases pod yield under intermittent drought in dry and hot atmospheric conditions but less so under wetter and cooler conditions in groundnut (*A. hypogaea* L.). Field Crops Research.

[ref-58] Xin Z, Aiken R, Burke J (2009). Genetic diversity of transpiration efficiency in sorghum. Field Crops Research.

[ref-59] Yau SK (2007). Winter versus spring sowing of rain-fed safflower in a semi-arid, high-elevation Mediterranean environment. European Journal of Agronomy.

[ref-60] Zaman-Allah M, Jenkinson DM, Vadez V (2011a). Chickpea genotypes contrasting for seed yield under terminal drought stress in the field differ for traits related to the control of water use. Functional Plant Biology.

[ref-61] Zaman-Allah M, Jenkinson DM, Vadez VA (2011b). Conservative pattern of water use, rather than deep or profuse rooting, is critical for the terminal drought tolerance of chickpea. Journal of Experimental Botany.

