# Supplementary material for: Safflower (Carthamus tinctorius L.) crop adaptation to residual moisture stress: conserved water use and canopy temperature modulation are better adaptive mechanisms
Source: PeerJ. 2023 Sep 11;11:e15928. doi: 10.7717/peerj.15928 (PMC10501382; doi:10.7717/peerj.15928)
Supplement: Supplemental Information 1 [file peerj-11-15928-s001.docx]

Supplementary fig 1. Climatic conditions from experimental plots during crop period i.e., post *rainy* 2020-21 & 2021-22.
