# Supplementary material for: Safflower (Carthamus tinctorius L.) crop adaptation to residual moisture stress: conserved water use and canopy temperature modulation are better adaptive mechanisms
Source: PeerJ. 2023 Sep 11;11:e15928. doi: 10.7717/peerj.15928 (PMC10501382; doi:10.7717/peerj.15928)
Supplement: Supplemental Information 2 [file peerj-11-15928-s002.doc]

**SUPPLEMENTARY FILES:**

**Supplementary table 1** List of genotypes selected for study

| Name | Type | Origin/ Developing centre |
| --- | --- | --- |
| GMU 2347 | Germplasm | Jalgaon, India |
| GMU 2648 | Germplasm | Jalgaon, India |
| GMU 3438 | Germplasm | Varanasi, India |
| GMU 3266 | Germplasm | Varanasi, India |
| GMU 2644 | Germplasm | Jalgaon, India |
| ISF 764 | Variety | Hyderabad, India |
| A 1 | Variety | Annigeri, India |
| Bhima | Variety | Solapur, India |
| PBNS 12 | Variety | Parbhani, India |
| NARI 6 | Variety | Phaltan, India |
| EC 523368-2  (GMU 7399) | Germplasm | Hyderabad, India |
| CO-1 | Variety | Coimbatore, India |

**Supplementary table 2** Physiological traits recorded in safflower genotypes under residual moisture conditions

|  |  | [SCMR](mailto:SCMR@45 DAS) | Transpiration rate (m mol H2O m-2 s-1­) | Stomatal conductance (m mol H2O m-2 s-1­) | Leaf temperature (oC) | Assimilation rate (µ CO2 m-2 s-2) | RWC  (%) | LAI | Days to I flower | Days to 50%  flower |
| --- | --- | --- | --- | --- | --- | --- | --- | --- | --- | --- |
| 1 | EC-523368-2 | 52.33 | 3.44 | 109.00 | 31.00 | 26.50 | 87.30 | 1.02 | 83.50 | 88.00 |
| 2 | A1 | 47.90 | 1.92 | 54.00 | 31.00 | 29.50 | 92.30 | 1.02 | 80.00 | 84.00 |
| 3 | BHIMA | 48.13 | 1.18 | 33.00 | 33.00 | 19.70 | 86.50 | 1.10 | 78.00 | 80.00 |
| 4 | CO-1 | 61.85 | 1.12 | 52.00 | 33.00 | 21.70 | 81.50 | 0.80 | 80.00 | 83.50 |
| 5 | GMU 2347 | 46.60 | 1.70 | 33.00 | 31.00 | 19.10 | 84.70 | 1.10 | 76.50 | 80.00 |
| 6 | GMU 2644 | 45.13 | 2.16 | 65.00 | 32.00 | 23.10 | 74.60 | 0.94 | 78.00 | 84.00 |
| 7 | GMU 2648 | 49.80 | 1.17 | 61.00 | 33.00 | 18.23 | 71.30 | 0.74 | 80.00 | 84.00 |
| 8 | GMU 3266 | 37.93 | 1.76 | 66.00 | 35.00 | 16.90 | 73.70 | 0.83 | 79.50 | 83.50 |
| 9 | GMU 3438 | 53.20 | 1.95 | 58.00 | 33.00 | 19.50 | 72.40 | 0.69 | 75.00 | 79.00 |
| 10 | ISF 764 | 51.40 | 1.85 | 108.00 | 35.00 | 19.30 | 76.10 | 1.13 | 78.00 | 82.50 |
| 11 | NARI 6 | 51.93 | 1.95 | 63.00 | 31.00 | 27.90 | 83.60 | 0.97 | 77.00 | 83.50 |
| 12 | PBNS 12 | 55.45 | 1.07 | 31.00 | 33.00 | 24.90 | 80.80 | 0.89 | 76.50 | 80.50 |
|  | Max value | 61.85 | 3.44 | 109.00 | 35.00 | 29.50 | 92.30 | 1.13 | 83.50 | 88.00 |
|  | Min Value | 37.93 | 1.07 | 31.00 | 31.00 | 16.90 | 71.30 | 0.69 | 75.00 | 79.00 |
|  | Average | 50.14 | 1.77 | 61.08 | 32.58 | 22.19 | 80.40 | 0.94 | 78.50 | 82.71 |
|  | STDEV | 5.88 | 0.65 | 25.52 | 1.44 | 4.13 | 6.74 | 0.15 | 2.26 | 2.49 |
|  | SE | 1.70 | 0.19 | 7.37 | 0.42 | 1.19 | 1.95 | 0.04 | 0.65 | 0.72 |
|  | CV | 0.12 | 0.37 | 0.42 | 0.04 | 0.19 | 0.08 | 0.16 | 0.03 | 0.03 |

SCMR: SPAD chlorophyll meter readings; RWC: Relative water content; LAI: Leaf area index. STDEV: Standard Deviation; SE: standard error: CV: Coefficient of variation.

**Supplementary table 3** Yield and attributing traits recorded from safflower genotypes under residual moisture conditions

|  |  | TDMH | NPC | NSC | WMC | WPC | WSC | SWP weYield | PH | HI |
| --- | --- | --- | --- | --- | --- | --- | --- | --- | --- | --- |
| 1 | EC-523368-2 | 32.60 | 7.00 | 7.60 | 1.51 | 10.36 | 5.89 | 8.82 | 56.40 | 0.27 |
| 2 | A1 | 50.90 | 6.10 | 8.30 | 2.26 | 10.33 | 7.04 | 18.64 | 76.50 | 0.37 |
| 3 | BHIMA | 93.20 | 14.30 | 33.10 | 3.23 | 23.82 | 29.96 | 12.74 | 80.00 | 0.14 |
| 4 | CO-1 | 46.00 | 7.50 | 7.50 | 2.38 | 11.53 | 6.80 | 12.21 | 87.40 | 0.27 |
| 5 | GMU 2347 | 34.20 | 7.20 | 10.70 | 1.56 | 9.00 | 5.53 | 11.94 | 68.20 | 0.35 |
| 6 | GMU 2644 | 41.60 | 7.90 | 11.60 | 2.58 | 13.15 | 10.57 | 9.04 | 72.50 | 0.22 |
| 7 | GMU 2648 | 34.80 | 6.60 | 9.90 | 2.38 | 10.72 | 9.06 | 11.10 | 70.00 | 0.32 |
| 8 | GMU 3266 | 44.00 | 7.00 | 9.00 | 1.94 | 10.77 | 8.12 | 13.16 | 69.50 | 0.30 |
| 9 | GMU 3438 | 50.90 | 9.10 | 13.40 | 2.03 | 15.32 | 13.07 | 15.84 | 70.50 | 0.31 |
| 10 | ISF 764 | 58.80 | 12.10 | 16.00 | 2.24 | 17.80 | 15.53 | 21.69 | 74.50 | 0.37 |
| 11 | NARI 6 | 37.10 | 7.30 | 12.80 | 1.64 | 7.94 | 8.34 | 8.51 | 91.10 | 0.23 |
| 12 | PBNS 12 | 56.00 | 11.80 | 18.30 | 2.81 | 17.42 | 19.63 | 17.96 | 74.80 | 0.32 |
|  | Max value | 93.20 | 14.30 | 33.10 | 3.23 | 23.82 | 29.96 | 21.69 | 91.10 | 0.37 |
|  | Min Value | 32.60 | 6.10 | 7.50 | 1.51 | 7.94 | 5.53 | 8.51 | 56.40 | 0.14 |
|  | Average | 48.34 | 8.66 | 13.18 | 2.21 | 13.18 | 11.63 | 13.47 | 74.28 | 0.29 |
|  | STDEV | 16.57 | 2.63 | 7.10 | 0.52 | 4.60 | 7.15 | 4.22 | 9.09 | 0.07 |
|  | SE | 4.78 | 0.76 | 2.05 | 0.15 | 1.33 | 2.07 | 1.22 | 2.62 | 0.02 |
|  | CV | 0.34 | 0.30 | 0.54 | 0.23 | 0.35 | 0.62 | 0.31 | 0.12 | 0.24 |

TDMH: Total dry matter at harvest(g); NPC: Number of primary capitulum; NSC: Number of secondary capitulum; WM: Weight of main capitulum(g); WPC: Weight of primary capitulum(g); WSC: Weight of secondary capitulum(g); SWP: Seed weight per plant(g); HI: Harvest index. STDEV: Standard Deviation; SE: standard error: CV: Coefficient of variation.
