# Supplementary material for: Safflower (Carthamus tinctorius L.) crop adaptation to residual moisture stress: conserved water use and canopy temperature modulation are better adaptive mechanisms
Source: PeerJ. 2023 Sep 11;11:e15928. doi: 10.7717/peerj.15928 (PMC10501382; doi:10.7717/peerj.15928)
Supplement: Supplemental Information 3 — Biomass, total dry matter at maturity stage; NPC, number of primary capitulum; NSC, number of secondary capitulum; WM, weight of main capitulum; WPC, weight of primary capitulum; SY, seed yield per plant; HI, harvest index. [file peerj-11-15928-s003.doc]

**SUPPLEMENTARY TABLE 2** Yield and yield attributes data of 12 safflower genotypes recorded under RSM conditions (2020-21, 22).

|  |  | Biomass | NPC | NSC | WMC | WPC | WSC | SY | plant height | HI |
| --- | --- | --- | --- | --- | --- | --- | --- | --- | --- | --- |
| 1 | EC-523368-2 | 32.60 | 7.00 | 7.60 | 1.51 | 10.36 | 5.89 | 8.82 | 56.40 | 0.27 |
| 2 | A1 | 50.90 | 6.10 | 8.30 | 2.26 | 10.33 | 7.04 | 18.64 | 76.50 | 0.37 |
| 3 | BHIMA | 93.20 | 14.30 | 33.10 | 3.23 | 23.82 | 29.96 | 12.74 | 80.00 | 0.14 |
| 4 | CO-1 | 46.00 | 7.50 | 7.50 | 2.38 | 11.53 | 6.80 | 12.21 | 87.40 | 0.27 |
| 5 | GMU 2347 | 34.20 | 7.20 | 10.70 | 1.56 | 9.00 | 5.53 | 11.94 | 68.20 | 0.35 |
| 6 | GMU 2644 | 41.60 | 7.90 | 11.60 | 2.58 | 13.15 | 10.57 | 9.04 | 72.50 | 0.22 |
| 7 | GMU 2648 | 34.80 | 6.60 | 9.90 | 2.38 | 10.72 | 9.06 | 11.10 | 70.00 | 0.32 |
| 8 | GMU 3266 | 44.00 | 7.00 | 9.00 | 1.94 | 10.77 | 8.12 | 13.16 | 69.50 | 0.30 |
| 9 | GMU 3438 | 50.90 | 9.10 | 13.40 | 2.03 | 15.32 | 13.07 | 15.84 | 70.50 | 0.31 |
| 10 | ISF 764 | 58.80 | 12.10 | 16.00 | 2.24 | 17.80 | 15.53 | 21.69 | 74.50 | 0.37 |
| 11 | NARI 6 | 37.10 | 7.30 | 12.80 | 1.64 | 7.94 | 8.34 | 8.51 | 91.10 | 0.23 |
| 12 | PBNS 12 | 56.00 | 11.80 | 18.30 | 2.81 | 17.42 | 19.63 | 17.96 | 74.80 | 0.32 |
|  | Max value | 93.20 | 14.30 | 33.10 | 3.23 | 23.82 | 29.96 | 21.69 | 91.10 | 0.37 |
|  | Min Value | 32.60 | 6.10 | 7.50 | 1.51 | 7.94 | 5.53 | 8.51 | 56.40 | 0.14 |
|  | Average | 48.34 | 8.66 | 13.18 | 2.21 | 13.18 | 11.63 | 13.47 | 74.28 | 0.29 |
|  | STDEV | 16.57 | 2.63 | 7.10 | 0.52 | 4.60 | 7.15 | 4.22 | 9.09 | 0.07 |
|  | SE | 4.78 | 0.76 | 2.05 | 0.15 | 1.33 | 2.07 | 1.22 | 2.62 | 0.02 |
|  | CV | 0.34 | 0.30 | 0.54 | 0.23 | 0.35 | 0.62 | 0.31 | 0.12 | 0.24 |

Biomass: Total Dry Matter at maturity stage, NPC: Number of primary capitulum, NSC: Number of secondary capitulum, WM: Weight of main capitulum, WPC: Weight of primary capitulum ,SY: Seed yield per plant, HI : Harvest Index.
