# Supplementary material for: Safflower (Carthamus tinctorius L.) crop adaptation to residual moisture stress: conserved water use and canopy temperature modulation are better adaptive mechanisms
Source: PeerJ. 2023 Sep 11;11:e15928. doi: 10.7717/peerj.15928 (PMC10501382; doi:10.7717/peerj.15928)
Supplement: Supplemental Information 4 — SCMR, SPAD chlorophyll meter readings; RWC, relative water content; LAI, leaf area index. STDEV, standard deviation; SE, standard error; CV, coefficient of variation. [file peerj-11-15928-s004.doc]

**SUPPLEMENTARY TABLE 3** Physiological and phenological data of 12 safflower genotypes recorded under RSM conditions (2020-21, 22).

|  |  | [SCMR](mailto:SCMR@45 DAS) | Transpiration rate  (mol H_2_O m^-2^ s^-1^) | Stomatal conductance  (m mol H_2_O m^-2^ s^-1^) | Leaf temperature (^o^C) | Assimilation rate (µ CO_2_ m^-2^ s^-2^) | RWC (%) | LAI | Days to I flower | Days to 50%  flower |
| --- | --- | --- | --- | --- | --- | --- | --- | --- | --- | --- |
| 1 | EC-523368-2 | 52.33 | 3.44 | 109.00 | 31.00 | 26.50 | 87.30 | 1.02 | 83.50 | 88.00 |
| 2 | A1 | 47.90 | 1.92 | 54.00 | 31.00 | 29.50 | 92.30 | 1.02 | 80.00 | 84.00 |
| 3 | BHIMA | 48.13 | 1.18 | 33.00 | 33.00 | 19.70 | 86.50 | 1.10 | 78.00 | 80.00 |
| 4 | CO-1 | 61.85 | 1.12 | 52.00 | 33.00 | 21.70 | 81.50 | 0.80 | 80.00 | 83.50 |
| 5 | GMU 2347 | 46.60 | 1.70 | 33.00 | 31.00 | 19.10 | 84.70 | 1.10 | 76.50 | 80.00 |
| 6 | GMU 2644 | 45.13 | 2.16 | 65.00 | 32.00 | 23.10 | 74.60 | 0.94 | 78.00 | 84.00 |
| 7 | GMU 2648 | 49.80 | 1.17 | 61.00 | 33.00 | 18.23 | 71.30 | 0.74 | 80.00 | 84.00 |
| 8 | GMU 3266 | 37.93 | 1.76 | 66.00 | 35.00 | 16.90 | 73.70 | 0.83 | 79.50 | 83.50 |
| 9 | GMU 3438 | 53.20 | 1.95 | 58.00 | 33.00 | 19.50 | 72.40 | 0.69 | 75.00 | 79.00 |
| 10 | ISF 764 | 51.40 | 1.85 | 108.00 | 35.00 | 19.30 | 76.10 | 1.13 | 78.00 | 82.50 |
| 11 | NARI 6 | 51.93 | 1.95 | 63.00 | 31.00 | 27.90 | 83.60 | 0.97 | 77.00 | 83.50 |
| 12 | PBNS 12 | 55.45 | 1.07 | 31.00 | 33.00 | 24.90 | 80.80 | 0.89 | 76.50 | 80.50 |
|  | Max value | 61.85 | 3.44 | 109.00 | 35.00 | 29.50 | 92.30 | 1.13 | 83.50 | 88.00 |
|  | Min Value | 37.93 | 1.07 | 31.00 | 31.00 | 16.90 | 71.30 | 0.69 | 75.00 | 79.00 |
|  | Average | 50.14 | 1.77 | 61.08 | 32.58 | 22.19 | 80.40 | 0.94 | 78.50 | 82.71 |
|  | STDEV | 5.88 | 0.65 | 25.52 | 1.44 | 4.13 | 6.74 | 0.15 | 2.26 | 2.49 |
|  | SE | 1.70 | 0.19 | 7.37 | 0.42 | 1.19 | 1.95 | 0.04 | 0.65 | 0.72 |
|  | CV | 0.12 | 0.37 | 0.42 | 0.04 | 0.19 | 0.08 | 0.16 | 0.03 | 0.03 |

SCMR: SPAD-Chlorophyll meter readings at 60 DAS, RWC: Relative water content, LAI: Leaf area index.
