# Supplementary material for: Safflower (Carthamus tinctorius L.) crop adaptation to residual moisture stress: conserved water use and canopy temperature modulation are better adaptive mechanisms
Source: PeerJ. 2023 Sep 11;11:e15928. doi: 10.7717/peerj.15928 (PMC10501382; doi:10.7717/peerj.15928)
Supplement: Supplemental Information 5 — SS, sum of squares; df, degrees of freedom; MS, mean sum of square; F, F value. [file peerj-11-15928-s005.docx]

**SUPPLEMENTARY TABLE 4** ANOVA emphasizing variance between the genotypes and the variance within genotypes.

| FTSW_NTR |  |  |  |  |  |  |
| --- | --- | --- | --- | --- | --- | --- |
| *Source of Variation* | *SS* | *df* | *MS* | *F* | *P-value* | *F crit* |
| GENOTYPE | 0.5555 | 11 | 0.0505 | 34.85641 | 4.64E-12 | 3.09** |
| ERROR | 0.034771 | 24 | 0.001449 |  |  |  |
|  |  |  |  |  |  |  |
| Total | 0.590271 | 35 |  |  |  |  |
|  |  |  |  |  |  |  |
| TE_WW |  |  |  |  |  |  |
| *Source of Variation* | *SS* | *df* | *MS* | *F* | *P-value* | *F crit* |
| GENOTYPE | 20.44408 | 11 | 1.858552 | 34.3813 | 5.4E-12 | 3.09** |
| ERROR | 1.297369 | 24 | 0.054057 |  |  |  |
|  |  |  |  |  |  |  |
| Total | 21.74144 | 35 |  |  |  |  |
|  |  |  |  |  |  |  |
| TE_WS |  |  |  |  |  |  |
| *Source of Variation* | *SS* | *df* | *MS* | *F* | *P-value* | *F crit* |
| GENOTYPE | 9.158485 | 11 | 0.83259 | 47.67096 | 1.4E-13 | 3.09** |
| ERROR | 0.419168 | 24 | 0.017465 |  |  |  |
|  |  |  |  |  |  |  |
| Total | 9.577653 | 35 |  |  |  |  |

SS: Sum of Squares, df: Degrees of freedom, MS: Mean sum of square, F: F value.
